# SNaPshot as a Valuable Option for the Identification of Mutations in Myeloma

**DOI:** 10.1016/j.ebiom.2014.11.014

**Published:** 2014-11-20

**Authors:** Jerome Moreaux

**Affiliations:** Department of Biological Haematology, CHU Montpellier, France; INSERM U1040, CHU Montpellier, Montpellier, France; University of Montpellier 1, UFR de Médecine, Montpellier, France

Multiple myeloma (MM) is a plasma–cell malignancy with a high degree of biological and genetic heterogeneity at presentation and a great variability in terms of clinical outcome in response to chemotherapy.

Over the last decades, the incorporation of novel agents (immunomodulatory drugs and proteasome inhibitors) with high-dose chemotherapy followed by autologous stem cell transplantation in eligible patients has improved the survival outcomes for myeloma patients (Barlogie et al., 2014).

Chromosomal abnormalities include recurrent 14q32 translocations (involving either *CCND1*, *MMSET* and *FGFR3*, *CCND3*, *c-MAF* or *MAFB*), hyperdiploidy, full or partial deletion of chromosomes 13 or 17 and 1q21 amplifications (Morgan et al., 2012). However, these chromosomal abnormalities are also observed in monoclonal gammopathy of unknown significance and genetic mutations have also been described as potent drivers of MM pathogenesis. Significant mutated genes were reported in MM including KRAS (23%), NRAS (20%), FAM46C (11%), TP53 (8%), DIS3 (11%), BRAF (6%), TRAF3 (5%) and PRDM1 (5%) (Chapman et al., 2011, Lohr et al., 2014). This heterogeneity is also translated at a subclonal level with a characterized complex clonal evolution during the progression of the disease (Bolli et al., 2014, Melchor et al., 2014). This high heterogeneity in MM emphasizes the requirement of tools for rapid identification of mutations constituting potent therapeutic targets in a personalized therapy approach.

O'Donnell and colleagues reported the interest of a Clinical Laboratory Improvement Amendments-approved, high-throughput, genotyping platform to determine the mutation status of a panel of known cancer genes in MM (O'Donnell et al., 2014). The method uses a highly sensitive multiplexed PCR-based assay to simultaneously identify 70 genetic loci frequently mutated in 15 cancer genes including NRAS, KRAS, TP53, BRAF and HRAS. The interest of their ready-to-use assay was investigated in 67 samples of patients with MM including a majority of samples collected at relapse. A candidate mutation was detected in 26 out of 67 tumor samples including KRAS (15/26), NRAS (6/26), TP53 (2/26), BRAF (2/26) and HRAS (1/26) mutations. Significant association between the occurrence of mutations and heavy-chain disease has been found. Interestingly, this methodology was performed on total bone marrow, without plasma cell purification, and displayed an overall sensitivity of 5% (requiring the presence of 10% clonal plasma cells within bone morrow). SNaPshot method is ready-to-use, faster and present an economic advantage compared to next generation sequencing in clinical practice. However, a limitation is linked to the fact that only already identified mutations can be investigated. Furthermore, addition of several genes described as frequently mutated in MM like DIS3, FAM46C, TRAF3 and PRDM1 could be beneficial to improve the assay.

The assay developed by O'Donnell et al. (2014) appears useful for rapid identification of mutations representing potential therapeutic targets in tumors with complex clonal evolution. Development of patient-specific personalized therapy may limit the side effects of treatment, improving compliance with dosing regimens and overall quality of life. The huge amounts of biological and genetic data generated by high-throughput technologies will facilitate pharmacogenomic progress, suggest novel druggable molecules, and support the design of future therapeutic strategies. Identification of BRAF activating mutation in MM has stimulated clinical exploration of BRAF inhibitors (Lohr et al., 2014, Andrulis et al., 2013). Recently, Andrulis et al. reported durable response in a patient harboring BRAF V600E mutation with relapsed MM refractory to all approved therapeutic options after treatment with vemurafenib (Andrulis et al., 2013). An open-label multi-center study investigating the efficacy and safety of vemurafenib in patients with BRAF mutation-positive cancers, including MM, is currently recruiting participants. Oncogenic mutations in MM could also be linked with response to therapy. More recently, Mulligan et al. demonstrated that NRAS mutation, but not KRAS, is associated with a significantly reduced sensitivity to single-agent bortezomib therapy as well as shorter time to progression in bortezomib-treated patients (Mulligan et al., 2014). Nevertheless, NRAS mutation did not impact outcome in patients treated with high-dose dexamethasone (Mulligan et al., 2014). This study underlines a clinical impact of NRAS mutation in MM.

The SNaPshot assay reported by O'Donnell and colleagues is undoubtedly useful in clinical practice (O'Donnell et al., 2014). This ready-to-use assay to detect major mutations in MM is an interesting method to integrate rapid genomic analysis into clinical routine for myeloma patients and could be valuable for adapting targeted treatment according to clonal evolution, during progression of the disease, in combination with existing therapies.

## Disclosure

The author declared that there are no conflicts of interest.
